# An improved autocidal gravid ovitrap for the control and surveillance of *Aedes aegypti*

**DOI:** 10.1186/1756-3305-6-225

**Published:** 2013-08-06

**Authors:** Andrew J Mackay, Manuel Amador, Roberto Barrera

**Affiliations:** 1 Entomology and Ecology Activity, Dengue Branch, Centers for Disease Control and Prevention, 1324 Calle Cañada, San Juan 00959, Puerto Rico; 2 Department of Entomology, University of Illinois, 320 Morrill Hall, 505 S. Goodwin Ave, Urbana, IL 61801, USA

**Keywords:** Aedes aegypti, Oviposition, Sticky ovitraps, Vector surveillance, Puerto Rico

## Abstract

**Background:**

Limited success has been achieved using traditional vector control methods to prevent the transmission of dengue viruses. Integrated control programs incorporating alternative tools, such as gravid ovitraps (lethal ovitraps and sticky ovitraps) may provide greater potential for monitoring and reducing vector populations and dengue virus transmission. We had developed an autocidal gravid ovitrap (AGO) as a simple, low-cost device for surveillance and control of *Ae. aegypti* without the use of pesticides that does not require servicing for an extended period of time. The purpose of our study was to improve the efficacy and efficiency of this device.

**Methods:**

Competitive assays were performed in the laboratory and an outdoor cage to evaluate whether modifications to the structure and appearance of our original trap design (AGO-A), and the addition of an olfactory bait (hay infusion), improve trap function. The performance of a modified trap design (AGO-B) was then assessed and compared with conventional ovitraps in a series of field tests in San Juan City, Puerto Rico. Generalized linear mixed models were used to analyze adult *Ae. aegypti* capture data from the laboratory, outdoor cage and field experiments.

**Results:**

Increasing the size of the trap entrance, altering the color of trap components, and increasing the volume/surface area of the aqueous bait significantly improved the performance of the AGO in the outdoor cage. In a subsequent field comparison, captures of *Ae. aegypti* females were 3.7 fold greater in the improved trap (AGO-B), compared with the original design (AGO-A). An infusion bait produced *“in situ”* significantly improved capture rates of the improved trap under both semi-natural and field conditions. Semi-weekly collections of *Ae. aegypti* females in the AGO-B were significantly correlated with cumulative rainfall 8 to 28 days prior to sampling, whereas egg collections in paired conventional ovitraps were not. When vector abundance was low, the AGO-B provided greater sensitivity and precision as a surveillance device, compared with paired conventional ovitraps.

**Conclusions:**

The AGO-B can be used to efficiently attract and capture gravid *Ae. aegypti* females for more than 8 weeks without the need for trap maintenance.

## Background

Access to practical tools for the surveillance and suppression of adult *Aedes aegypti* L. is critical to developing an effective and sustainable dengue vector control program [1,2]. Surveillance of *Ae. aegypti* is primarily limited to sampling its immature stages. Little effort is made to directly reduce the survival of adult vectors at a community scale, except for truck-mounted, ultra-low volume applications of insecticides in public and peridomestic spaces, which may have limited efficacy against *Ae. aegypti*[3-6]. There is a need to identify alternative tools and strategies for cost-effective control and surveillance of adult vectors.

The ovitrap has been used for many decades as a sensitive, inexpensive, passive surveillance tool for detecting the presence of container-inhabiting mosquitoes, and for providing a relative measure of temporal changes in adult abundance. The addition of a larvicide or autocidal mechanism allows long-term use of the ovitrap with minimal risk of the device becoming a productive source of adult mosquitoes (ie., autocidal ovitrap, eg. [7,8]). Ovitraps can also be modified to collect gravid females by incorporating an adhesive capture surface (sticky ovitraps; eg. [9]). Adult mosquitoes collected in sticky ovitraps provide a direct measure of adult abundance, may be identified by morphology in the field, and can be processed for the detection of pathogens or host blood meal identification [10-12]. Sampling with sticky ovitraps is a more sensitive method for detecting the presence of *Ae. aegypti* in comparison with sampling of immatures [13,14]. Though they tend to capture fewer mosquitoes than BG-sentinel traps [15] and provide a less representative sample of the adult population than backpack aspirators [16], sticky ovitraps can be a more practical and affordable alternative for routine vector surveillance. Indices based on the number of gravid females collected in sticky traps have been used to describe the spatial-temporal distribution of *Ae. aegypti* and dengue virus (DENV) transmission risk [10,14,17-19].

In addition to their value for vector surveillance, various ovitrap devices have been evaluated as tools for suppressing *Ae. aegypti* populations [7,8,20,21]. Lethal ovitraps, which contain an oviposition substrate treated with a residual insecticide (eg., [22]), and sticky ovitraps eliminate gravid female mosquitoes as they attempt to oviposit inside the trap (both referred to collectively hereafter as “gravid ovitraps”; GOs), thereby reducing the daily survival rate of the fraction of the adult vector population most likely to be infective and potentially lowering vectorial capacity. A potential disadvantage of using an insecticide rather than an adhesive in a GO is that efficacy could be compromised when the device is used against a vector population with a low susceptibility to the insecticide class.

A potential limitation of using ovitrap devices for vector control is their ability to compete with existing container habitats in the environment [20,23,24]. It has been recommended that trap deployment should be preceded by a community-wide source reduction effort to mitigate this effect [20,25,26]. Furthermore, variability in the abundance and quality of alternative aquatic habitats in time and space likely diminishes the precision of ovitraps to estimate adult abundance [27]. Negative effects of competing oviposition sites on trap efficacy might be lessened by increasing the relative attractiveness of the ovitrap device. Typically ovitraps are constructed from relatively small containers (capacity 0.5-2 l). Previous studies have reported a positive association between container size, and the frequency of positivity and the number of eggs deposited by *Ae. aegypti*[28-30]. A larger ovitrap may provide a more conspicuous visual target to gravid females searching for suitable oviposition sites, and a greater release rate of water vapor and other volatile attractants, potentially extending the range of attractive olfactory stimuli released by a trap. Ovitrap devices with a larger capacity may also be less susceptible to failure due to desiccation of the aqueous attractant.

Aqueous infusions of decaying botanical material, prepared either prior to trap deployment or *in situ*, and synthetic blends of compounds identified from infusions of decaying plants, have been shown to significantly increase the number of *Ae. aegypti* eggs collected in ovitraps under field conditions [31-33], and therefore are often used as baits in GOs [20,34,35]. However, the relative contributions of olfactory attraction and contact-mediated oviposition stimulants to enhanced oviposition in infusion baited traps are unclear. Field studies comparing numbers of adult *Ae. aegypti* captured in sticky ovitraps baited with water and plant-based infusions have not shown these baits to enhance attraction to the trap [16,36].

The objective in this study was to improve upon an autocidal gravid ovitrap device (AGO-A) that we had developed as a low-cost and practical device for routine surveillance and control of *Ae. aegypti* mosquitoes. In a previous area-wide, integrated vector control intervention (source reduction and larviciding) in two communities in southern Puerto Rico, inclusion of the AGO-A as part of the intervention resulted in a significantly greater reduction (≈43%) in the relative abundance of parous and gravid *Ae. aegypti*, compared with the community where only source reduction and larviciding was performed (CDC, unpublished data). Following these encouraging results, we evaluated several modifications intended to improve the efficacy and efficiency of the trap. We also evaluated the contribution of an olfactory bait (hay infusion generated *in situ*) to trap performance over an extended use period (> 6 weeks). In this paper we describe how our improved, autocidal gravid ovitrap design (AGO-B) was developed and tested under laboratory and field conditions in Puerto Rico.

## Methods

### Gravid mosquitoes

Gravid females used in behavioral assays under laboratory and semi-natural conditions were from the F2 to F5 generations of colonies established from eggs collected from Patillas, Puerto Rico (18° 0′ 27″ N, 66° 0′ 35″ W). Individual cohorts were reared at 26 ± 1.0 °C, 65–80 percent relative humidity, and a photoperiod of 12:12 (L:D), in pans containing 150 larvae and 1 liter of dechlorinated water, and fed increasing amounts of finely ground rabbit chow during development (0.7-1.6 mg per larva per day). A minimum of 3 days after adult emergence (range 3-7d), females were artificially fed on bovine or porcine blood daily for five consecutive days, then maintained for an additional 3 days prior to use in behavioral assays (range 10-14d post-emergence). A cotton wick provided 10 percent sucrose solution from adult emergence to 1 day prior to the 1st day of blood feeding, and from the 4th day of blood feeding to the day prior to use.

### Competitive assays under semi-natural conditions

Trap designs and infusion baits were compared in competitive assays under semi-natural conditions in two field cages. Most of these experiments were performed in Cage A, a 10 m diameter, 3.4 m high geodesic dome tent (Shelter Systems, Santa Cruz, California, USA), with two 0.2 × 1.5 m and four 1.5 × 1.5 m screen windows providing ventilation. Traps were arranged at fixed locations in a ring 1.6 m from the wall of the field cage, with a minimum distance of 2.6 m (6 or 8 traps) or 4.8 m (3 or 4 traps) between traps. A single experiment (attraction to anaerobically fermented hay infusion in the AGO-B; see below) was conducted in a smaller, rectangular field enclosure (Cage B; 7.0 × 2.7 × 3.7 m) with screen walls on two adjacent sides. In Cage B, a trap was assigned to each of two locations approximately 2 m apart in the center of the enclosure.

In each trial, 120 (Cage B) or 150 (Cage A) gravid *Ae. aegypti* females were released from the center of the arena. Experiments were conducted from 2.5 hrs prior to sunset, and traps were recovered 2.5 hrs after sunrise, corresponding to the diel rhythm of oviposition activity of gravid *Ae. aegypti* females [37]. A sweep net and BG-sentinel trap (operated for 6 hours) were used to collect females remaining in the cage after each trial.

Data from the competitive assays in the screened room and field cage were analyzed by generalized linear mixed models (GLMMs) with repeated measures (SPSS ver. 12.0, SPSS, Inc., Chicago, Illinois, USA), with number of females captured as the response variable, treatment as a fixed effect, and trap location (subject) and trial (repeated measure) as random effects. Results are reported as marginal means with 95 percent confidence intervals. Bonferroni adjustments were made for assays comparing more than two treatments.

### Autocidal gravid ovitraps

**Figure 1:**
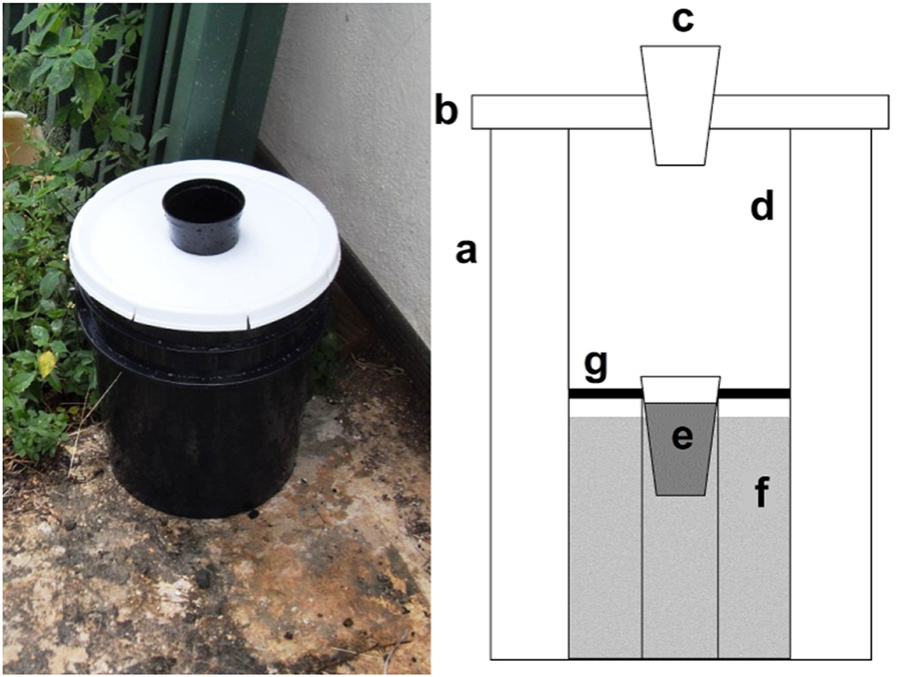
**The original autocidal gravid ovitrap (AGO-A).** Components include a 19 l black pail **(a)**, a white pail lid **(b)**, an 8.8 cm entrance diameter **(c)** a white capture surface (CS) coated with adhesive **(d)**, PAM **(e)**, a 2.5 l capacity infusion reservoir **(f)**, and a screen barrier between the CS and the infusion reservoir **(g)**.

The initial trap design (AGO-A; Figure 1) consisted of seven basic components: (i) black polyethylene pail (19 l volume) (ii) white pail lid, (iii) black plastic cup with the bottom removed (8.8 cm diameter at upper opening, 11 cm height) that served as the trap entrance, (iv) cylindrical capture chamber immediately below the trap entrance formed by a white, styrene cylinder (16 cm diameter); the inner surface (capture surface; CS) coated with 155 g / m^2^ of a non-setting, polybutylene adhesive (32UVR, Atlantic Paste & Glue Co., Inc., Brooklyn, New York, USA), (v) autocidal oviposition substrate (polyacrylamide co-polymer hydrogel; “PAM”) at the base of the capture chamber, (vi) reservoir (3.8 l black, polyethylene bucket) for the infusion attractant immediately below the capture chamber, and (vii) screen barrier preventing adult mosquitoes from moving between the capture chamber and the infusion reservoir. Drainage holes in the top of the bucket and bottom of the pail allowed excess infusion to drain from the trap (maximum infusion capacity 2.5 l).

**Figure 2:**
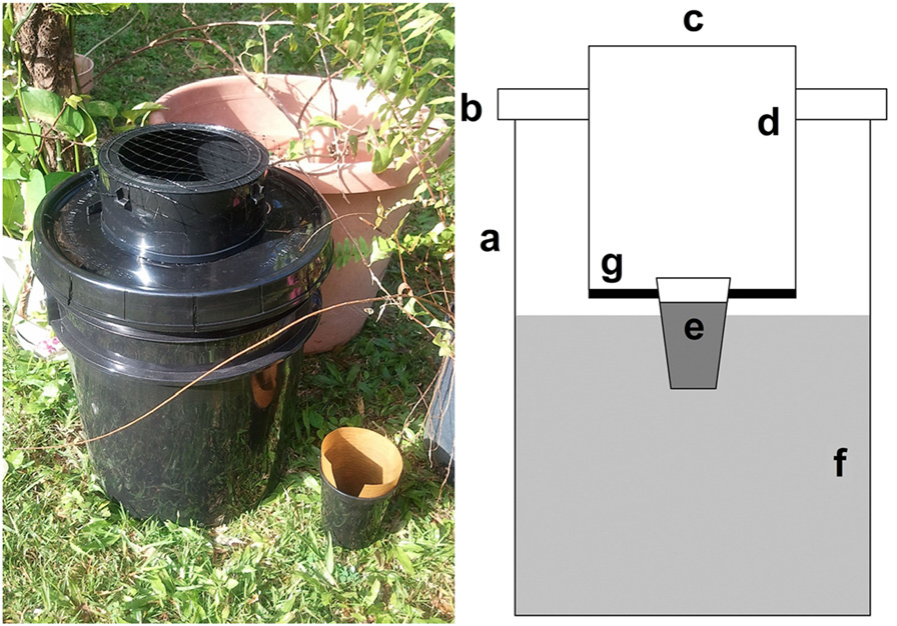
**The improved autocidal gravid ovitrap (AGO-B).** Components include a 19 l black pail **(a)**, a black pail lid **(b)**, a 12.8 cm entrance diameter **(c)**, a black capture surface (CS) coated with adhesive **(d)**, PAM **(e)**, a 9.3 l capacity infusion reservoir **(f)**, and a screen barrier between the CS and the infusion reservoir **(g)**. A conventional ovitrap is visible in the foreground of the photograph, on the right-hand side of the AGO-B.

The AGO-B (Figure 2) was constructed similar to the AGO-A, except for the following changes. The color of the pail lid was changed from white to black. To form the capture chamber, the 3.8 l black, polyethylene bucket was inverted and raised so that it transected the pail lid. A 12.8 cm diameter hole was cut in the apex of the capture chamber to create the trap entrance. The color of the adhesive-coated CS lining the capture chamber was changed from white to black. The infusion attractant was added directly to the pail to a maximum volume of 9.3 l. The trap entrance was covered by ¾” black polypropylene, oriented netting (Industrial Netting, Minneapolis, Minnesota, USA) to exclude the entry of larger debris or organisms.

### Development of the AGO-B

The transition from the initial trap design (AGO-A) to the final model (AGO-B) was directed by the results of a series of competitive assays conducted under semi-natural conditions (Cage A) assessing the influence of changes to the structure and appearance of the trap on the number of gravid females captured. In each experiment, traps were baited with a 1:1 dilution of an anaerobically-fermented (AF) hay infusion prepared in a closed container similar to the method of [31], except that the concentration of hay was reduced to 3.8 g per l of water. For each trial, 61 g of *Cynodon nlemfuensis* Vanderyst (Bogdan) was added to 16 l of dechlorinated tap water in a sealed (19 l capacity) plastic container. Infusions were aged for 7 days in a shaded location outdoors under ambient conditions, then sieved with a 1.2 mm mesh prior to use, and diluted with dechlorinated tap water (1:1). The diluted infusion was added to the infusion reservoir to ≈ 80 percent of maximum capacity (AGO-A = 2 l; AGO-B = 8 l). The autocidal oviposition substrate (PAM) was prepared by hydrating Outdoor Variety Plant Gel crystals (DNB Designs, Inc., Colorado Springs, Colorado, USA) with undiluted AF infusion (1 g / 100 ml infusion) for a minimum of 3 hours prior to each experiment. The PAM acts as an attractive surface for oviposition while preventing immature development (CDC, unpublished data).

In the first test, the effects of trap entrance diameter (ED) and the color of the CS on trap performance were examined. The numbers of gravid *Ae. aegypti* females captured in the AGO-A (8.8 cm ED + white CS) were compared with captures in three modified AGO-A designs; (i) 8.8 cm ED + black CS, (ii) 12 cm ED + white CS, and (iii) 12 cm ED + black CS. A black, plastic truncated cone, of identical height (11 cm) and similar shape as the 8.8 cm ED cup, was used to create the entrance of trap designs (ii) and (iii). In each of 6 trials, two traps representing each treatment were randomly assigned to eight fixed locations in the field cage.

The second experiment assessed the combined effects of altering the shape of the trap entrance, the position of the CS, and the size of the infusion reservoir (volume and surface area of infusion) on trap performance by comparing capture rates between the AGO-B (but with a white pail lid and without a ¾” exclusion screen) and the modified AGO-A design (iii) from the previous experiment (12 cm ED, black CS). For each of three trials, four traps of each type were randomly distributed among 8 fixed locations in the field cage.

In the third experiment, the influence of a color contrast between the trap entrance and the pail lid on the performance of the AGO-B was examined. Two AGO-Bs with a white lid, and two AGO-Bs with a black lid, were randomly placed at 4 fixed locations in each of eight trials. The ¾” exclusion screen was not used in this test.

The fourth experiment was performed to test whether capture efficiency is reduced by the use of an exclusion screen to prevent larger organisms or debris (leaves, etc.) from entering the trap. In each of three trials, AGO-Bs were placed at eight fixed locations, and a ¾” exclusion screen was used to cover the trap entrance of four randomly selected traps.

### Attractiveness of hay infusion bait produced in situ

Two initial experiments were performed under semi-natural conditions to verify that the use of “standard” AF infusion bait significantly enhances attraction to both AGO models. In the first test, AGO-As were baited with either tap water or a 1:1 dilution of AF infusion, prepared as described previously. In each of five trials, four AGO-As representing each treatment group were randomly assigned to one of eight fixed locations in Cage A. This experiment was repeated in Cage B using the AGO-B. Eight replicate trials were performed in the second experiment.

After verifying that the “standard” AF infusion bait enhances the capture of gravid *Ae. aegypti* females in both trap designs, three sets of experiments were performed in Cage A to assess the attractiveness of hay infusion fermented *in situ* by adding a packet of dry hay to the infusion reservoir filled to ≈ 80 percent of maximum capacity with dechlorinated tap water (3.8 g hay / l of water). Hay packets were prepared by folding the dry hay into a compact form and wrapping it with a plastic cable tie.

In the first experiment, the relative attractiveness of infusion produced *in situ* using a hay packet, in both the AGO-A and the AGO-B, was compared with our standard bait (1:1 dilution of AF infusion). The AF and *in situ* infusions were prepared as described previously. After aging for 7 days, each infusion was removed from its respective receptacle, sieved and 1.5 l was transferred to a 3.8 l plastic container, which was placed inside an AGO-B (no additional water or infusion was added to the infusion reservoir). For each trial (n = 6), an AGO-B representing each infusion type was randomly assigned to one of three fixed locations in the field cage.

The second test of an *in situ* infusion evaluated the influence of hay concentration on attraction to the AGO-B. Traps were baited with 8 l of tap water and either 3.8 g or 8.3 g of hay per l (higher concentration from [31]), then aged under natural conditions in a shaded location. Assays were performed at 7 and 28 days, with three replicate trials performed at each time point. In each trial, four AGO-Bs with the low hay concentration, and four AGO-Bs with the high hay concentration were randomly assigned to one of the eight fixed locations in the field cage. The numbers of gravid females released per trial was reduced to 120 in the first time point (7 days).

It was observed in both the laboratory and field tests that gravid females will often expel their eggs when they are trapped on the CS of the AGO-A and AGO-B (“death stress oviposition”; [38]). We were concerned that odors released by con-specific larvae in the infusion could increase attraction to the trap, resulting in a potential sampling bias (ie. relative attractiveness of trap positively correlated with number of females previously captured). To investigate this, AGO-Bs baited with an *in situ* infusion (30g hay packet) were aged under field conditions for 7 days in a shaded location. Five days prior to testing, 0, 50 or 500 first instar *Ae. aegypti* larvae were added to the infusion reservoir of each trap. For each trial (n = 9), a single AGO-B representing each larval density was randomly assigned to one of three fixed locations.

### Field assessments of the AGO-B

Three consecutive field experiments evaluating the performance of the AGO-B were conducted in 2011 in the San Juan metropolitan area, Puerto Rico. The study site (“El Comandante”) was a residential neighborhood approximately 1.5 km^2^ in size (described in [39]). The 30 residential properties selected for trap placement were uniformly distributed, with an average minimum distance of 145 m between selected properties. At each selected property, 2 AGOs (1 per treatment) were placed at fixed positions that provided some protection from direct sunlight (ie. next to the home or vegetation), and were at least 5 m apart, on opposite sides of the property when possible. Every Thursday and Monday, adult mosquitoes were removed from the CSs and enumerated by species and sex, and the positions of the two treatments were rotated within each property. To facilitate consistent 3 day sampling intervals, the trap entrance was sealed with window screens from Thursday to Friday to prevent entry of mosquitoes. A pair of conventional ovitraps (“enhanced pairs” of [31]) were placed at a home adjacent to each selected property to provide an additional measure of *Ae. aegypti* activity in the study area. Ovitraps were replaced at the start of each 3 day sampling period. Meteorological data was collected at the Luis-Muñoz International Airport, approximately 4 km from the study site.

In the first field experiment, numbers of adult mosquitoes captured in the original trap model (AGO-A) and the improved device (AGO-B) were compared from 15 February to 18 April. Traps, constructed as described previously, were baited with a 30 g hay packet (*in situ* infusion) and distributed among the 30 selected properties. One property was later excluded from the experiment after home renovations prevented access to the traps.

The second field experiment (18 April to 12 May) was initiated immediately following the first experiment to assess whether restoration of attraction to the AGO-B after extended use requires a full replacement of the *in situ* infusion bait, or if it would be sufficient to replace the hay packet and the water lost via evaporation, but retain the fluid remaining in the infusion reservoir (partial replacement). Fresh traps (designated AGO-B) were prepared as described above to represent a “full bait replacement”. The “partial bait replacement” condition was generated by replacing the CSs, PAM and hay packets in the AGO-B devices used in the previous experiment (now designated AGO-Bp). Tap water was added to each AGO-Bp to bring the volume up to maximum capacity (9.3 l).

In the third field experiment (6 June to 21 July), we examined the relative contribution of the *in situ* infusion to trap performance during extended use (> 6 weeks) by comparing adult captures in traps baited with a hay packet (AGO-B) and traps without a hay packet (only water; AGO-Bw). Samples were collected from AGOs at all 30 selected properties in this experiment.

A GLMM with a Poisson distribution and log link function was constructed for each field experiment to evaluate the influence of AGO trap design (fixed effect) on the number of *Ae. aegypti* females collected per 3 day period (SPSS, Inc.). Trap location within each home was included as a random effect. Covariates evaluated in each model included cumulative rainfall and average daily mean air temperature 1 to 7, 1 to 21, 1 to 28, 8 to 21 and 8 to 28 days preceding the sampling period. These time intervals were chosen based on previously observed temporal relationships between the abundance of *Ae. aegypti* females (measured by BG-sentinel sampling), and rainfall and temperature and at the same study site [40]. The −2 log likelihood ratios of nested models were compared to identify the best fit model for each experiment.

### Optimal sample size calculations for the AGO-B and paired ovitraps

Data collected from the field experiments were used to calculate the optimal number of AGO-B devices required for estimating the relative abundance of *Ae. aegypti* adult females. Taylor’s Power Law equations (log_10_*s*^2^ = log_10_ (*a*) + *b* log_10_ (*m*); [41]) were used to regress the log_10_ transformed variance (*s*^2^) and mean values (*m*) for each sampling date, in each field experiment, for both devices (GraphPad Prism version 5.04 for Windows, GraphPad Software, San Diego, California, USA). Regression coefficients from individual experiments were compared [42], and data from experiments with coefficients that were not significantly different were pooled. Coefficients of a Taylor’s Power Law regression model of the pooled data set were used to calculate the minimum sample size (*N*) required to estimate female *Ae. aegypti* relative abundance at a range of precision levels (0.1, 0.2, 0.3) using the formula: *N* = (*Z*_α/2_ / *D*)^2^ ⋅ *am*^(*b*-2)^, where *Z*_α/2_ is the upper α/2 part of the standard normal distribution, *D* is the specified precision level (fixed proportion of the mean) and *m* is the expected number of specimens collected per trap per day [43]. Identical analyses were performed with data collected in the paired ovitraps to estimate the optimal number of conventional ovitrap pairs required for sampling eggs. To directly compare estimated minimum sample sizes between the two sampling devices, a major axis regression was used to model the relationship between the mean adult collections in the AGO-B and mean egg collections in the paired ovitraps (SPSS, Inc.).

### Long-term efficacy of the AGO-B

At the conclusion of field experiment 1 (62 days), we conducted a census of the condition of AGO-Bs, and the volume of infusion remaining in each trap was measured. It was observed that the CS of the AGO-B becomes coated with many small flies, dust and other debris after extended use. Used CSs from twelve randomly selected AGO-Bs were brought back to the laboratory to test whether their ability to capture gravid females had diminished after extended use.

To test whether the efficacy of the adhesive was reduced, a 2-way competitive assay was conducted under laboratory conditions (≈26°C, 70% RH, 12:12 L:D) in a 3.8 × 2.7 × 2.9 m room. In each of 4 trials, 3 AGO-Bs with a fresh CS, and 3 AGO-Bs with a 62 day old CS (from field experiment 1) were used. Traps were randomly assigned to 6 fixed locations forming a 2 × 3 m grid. One hundred and twenty gravid *Ae. aegypti* females were released from the center of the room 3 hours prior to the start of the scotophase, and the numbers captured in each trap were determined 20 hours later. Eggs deposited on the PAM were also collected and counted. The numbers of adults and eggs collected in each trap were compared as described previously for the field cage tests. A square root transformation of the egg data was used to approximate a normal distribution.

## Results

### Development of the AGO-B

**Figure 3:**
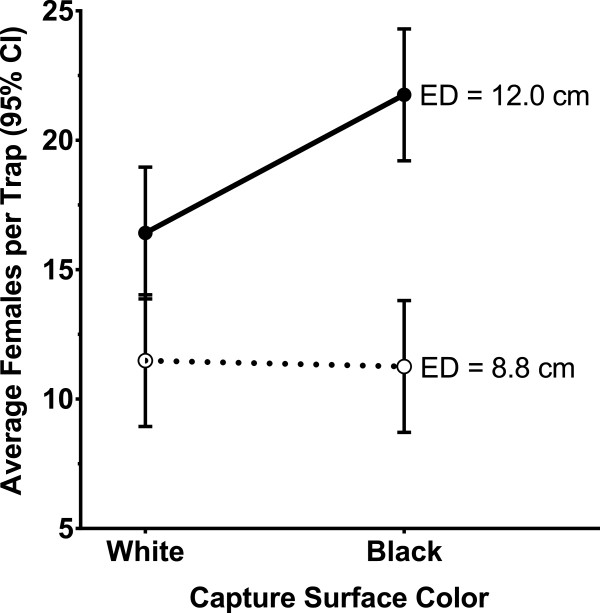
Influence of entrance diameter and capture surface color on AGO performance under semi-natural conditions.

Greater numbers of females were captured in the AGO-A with the black CS and large ED (Figure 3). The interaction between trap ED and CS color (F = 5.0, *P* < 0.05) had a significant influence on the numbers of adults captured. In the trap with the smaller ED, no difference in efficacy was observed between traps with a white CS and traps with a black CS.

When the modified AGO-A design (12 cm ED and black CS) was compared with the AGO-B in competitive assays in the field cage, trap design was found to significantly influence capture efficiency (F = 21.0, *P* < 0.001). Mean (95% CI) numbers of females captured per trap in competitive assays in the field cage were 13.1 (10.9, 15.3) and 20.0 (17.8, 22.2) in the modified AGO-A and the AGO-B designs, respectively.

Lid color had a significant influence on trap captures in the AGO-B (F = 11.3, *P* < 0.01). The mean (95% CI) number of females captured in traps with a black lid was 37.4 (34.0, 40.9), compared with 29.5 (26.0, 32.9) in traps with a white lid.

In the field cage, the addition of a ¾” exclusion screen to the trap entrance had a significant positive effect on the capture of female *Ae. aegypti* in the AGO-B (F = 6.1, *P* < 0.05). Mean (95% CI) numbers of females captured in traps with and without an exclusion screen were 20.2 (17.7, 22.8) and 16.2 (13.7, 18.8), respectively.

### Attractiveness of hay infusion bait produced in situ

In both trap designs (AGO-A, AGO-B), a 1:1 dilution of AF infusion bait resulted in a significantly greater number of females captured under semi-natural conditions (Table 1). Fermentation conditions of the infusion bait had a significant influence on attraction to the AGO-B (Table 2); greater numbers of females were captured in traps baited with an infusion aged in a closed container (anaerobic), compared with traps where the infusion was aged *in situ*. Neither the mass of hay used to produce infusion *in situ*, nor the presence of conspecific larvae in the hay infusion significantly influenced attraction to the AGO-B in the field cage (Table 2).

**Table 1:** Influence of infusion on attraction to AGOs

**Trap design**	**Attractant**	**Avg. females per trap (95% CI)**	**F**	***P*-value**
AGO-A	Tap water	7.5 (4.1, 10.9)	22.8	<0.001
	Anaerobically-fermented infusion	18.8 (15.4, 22.2)		
AGO-B	Tap water	33.0 (16.2, 49.8)	9.4	<0.05
	Anaerobically-fermented infusion	62.6 (45.9, 79.4)		

**Table 2:** Influence of ageing-conditions, hay concentration and conspecific odors on attraction to infusion-baited AGO-Bs

**Experiment**	**Infusion age (days)**	**Infusion attractant**	**Avg. females per trap (95% CI)**	**F**	***P*-value**^**b**^
1 - Infusion fermentation conditions	7	Anaerobically-fermented	46.1 (40.7, 51.6)	5.1	<0.05
		*In situ* (AGO-A)	37.4 (31.9, 42.8)		
		*In situ* (AGO-B)	36.3 (30.9, 41.7)		
2 - Hay concentration^a^	7	*In situ* (1×)	12.1 (9.6, 14.6)	0.8	NS
		*In situ* (2.2×)	10.1 (8.1, 13.1)		
	28	*In situ* (1×)	19.4 (17.0, 21.7)	3.8	NS
		*In situ* (2.2×)	16.2 (13.8, 18.6)		
3 - Addition of conspecific larvae	7	*In situ*	34.9 (29.1, 40.6)	1.5	NS
		*In situ* + 50 larvae	39.7 (33.9, 45.4)		
		*In situ* + 500 larvae	41.1 (35.4, 46.9)		

^a^ 1× infusion concentration = 3.8 g of hay per l of dechlorinated tap water, 2.2× infusion concentration = 8.3 g of hay per l of dechlorinated tap water. A 1× concentration was used to prepare *in situ* infusions in experiments 1 and 3.

^b^ NS: not significant (*P* ≥ 0.05).

### Field assessments of the AGO-B

**Figure 4:**
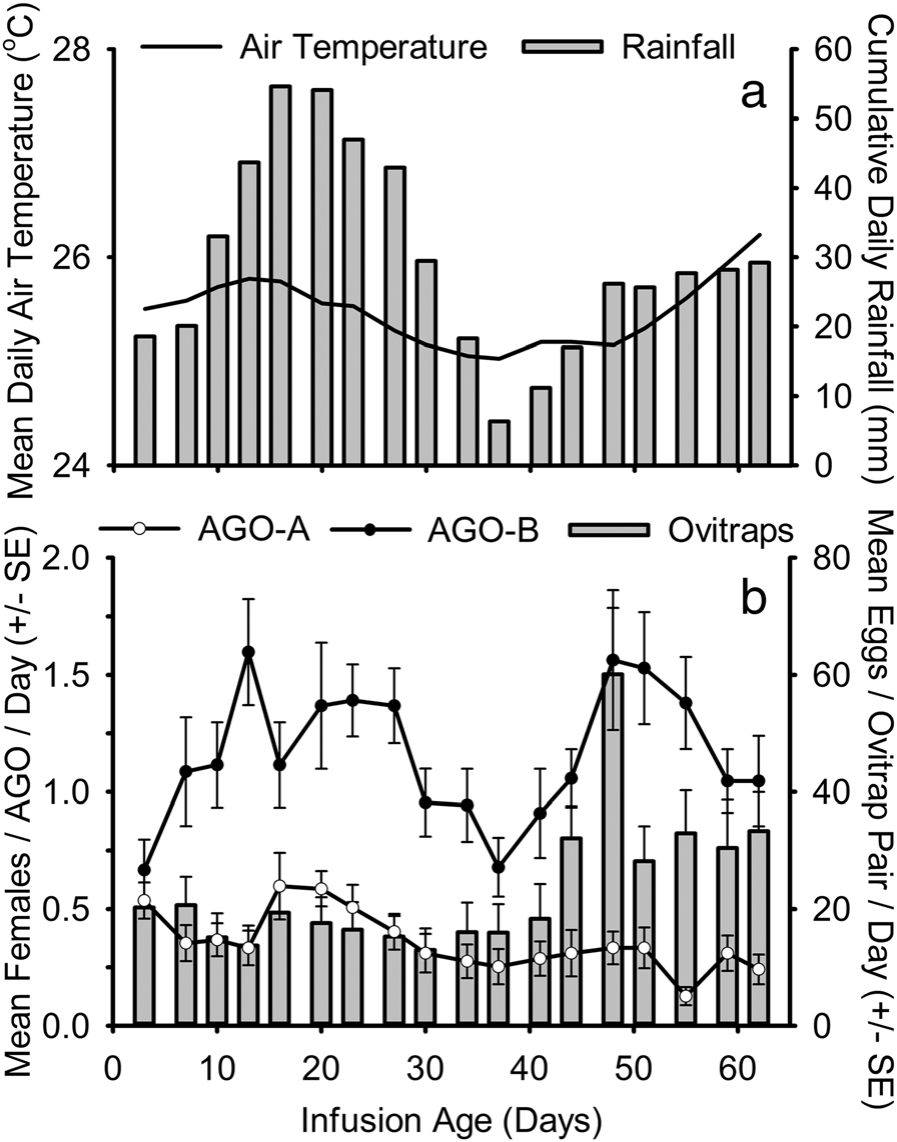
**Field comparison of original (AGO-A) and improved (AGO-B) trap designs.** Cumulative daily rainfall and average daily mean air temperature 8 to 28 days preceding sampling **(a)**, and the average numbers of *Aedes* eggs collected in ovitrap pairs and *Aedes aegypti* adult females collected in AGOs **(b)**, from 15 February to 18 April, 2011 (Field experiment 1).

**Figure 5:**
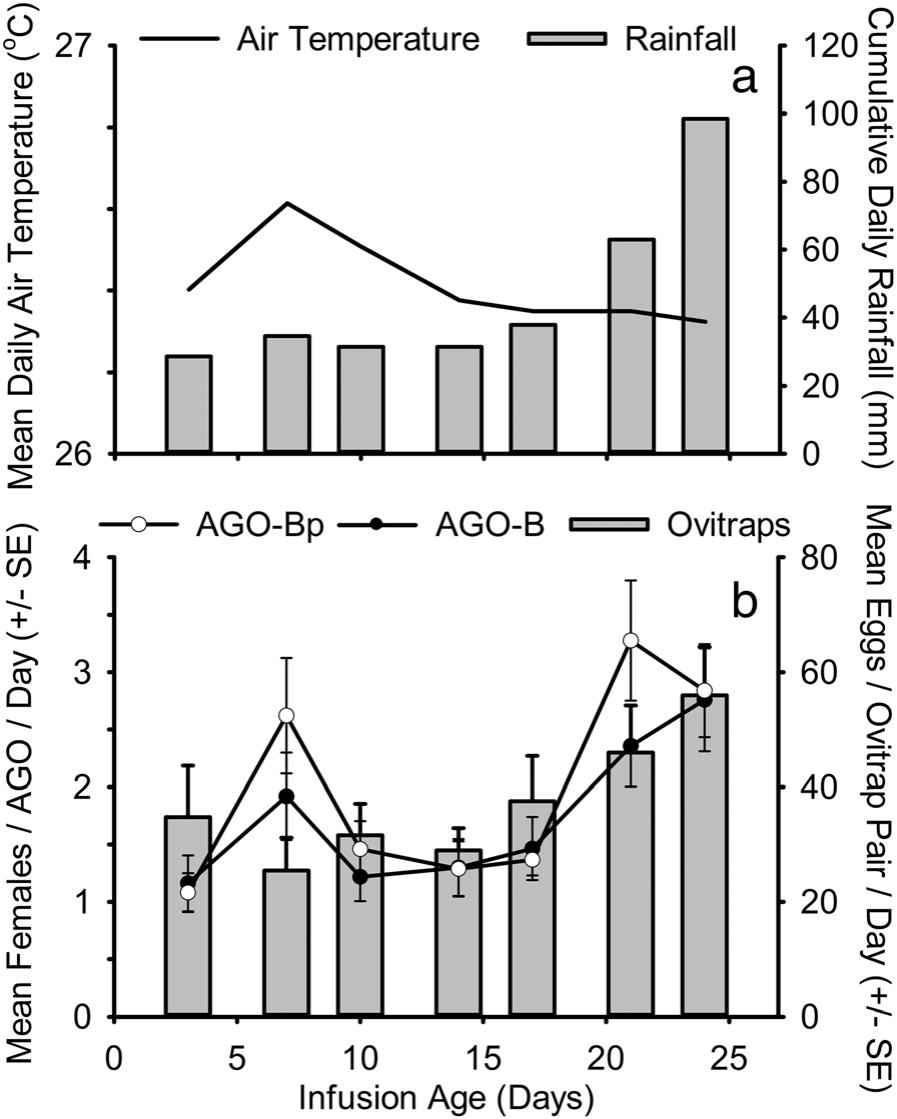
**Field comparison of the improved trap with full (AGO-B) or partial (AGO-Bp) infusion bait replacement.** Cumulative daily rainfall and average daily mean air temperature 8 to 28 days preceding sampling **(a)**, and the average numbers of *Aedes* eggs collected in ovitrap pairs and *Aedes aegypti* adult females collected in AGOs **(b)**, from 18 April to 12 May, 2011 (Field experiment 2).

**Figure 6:**
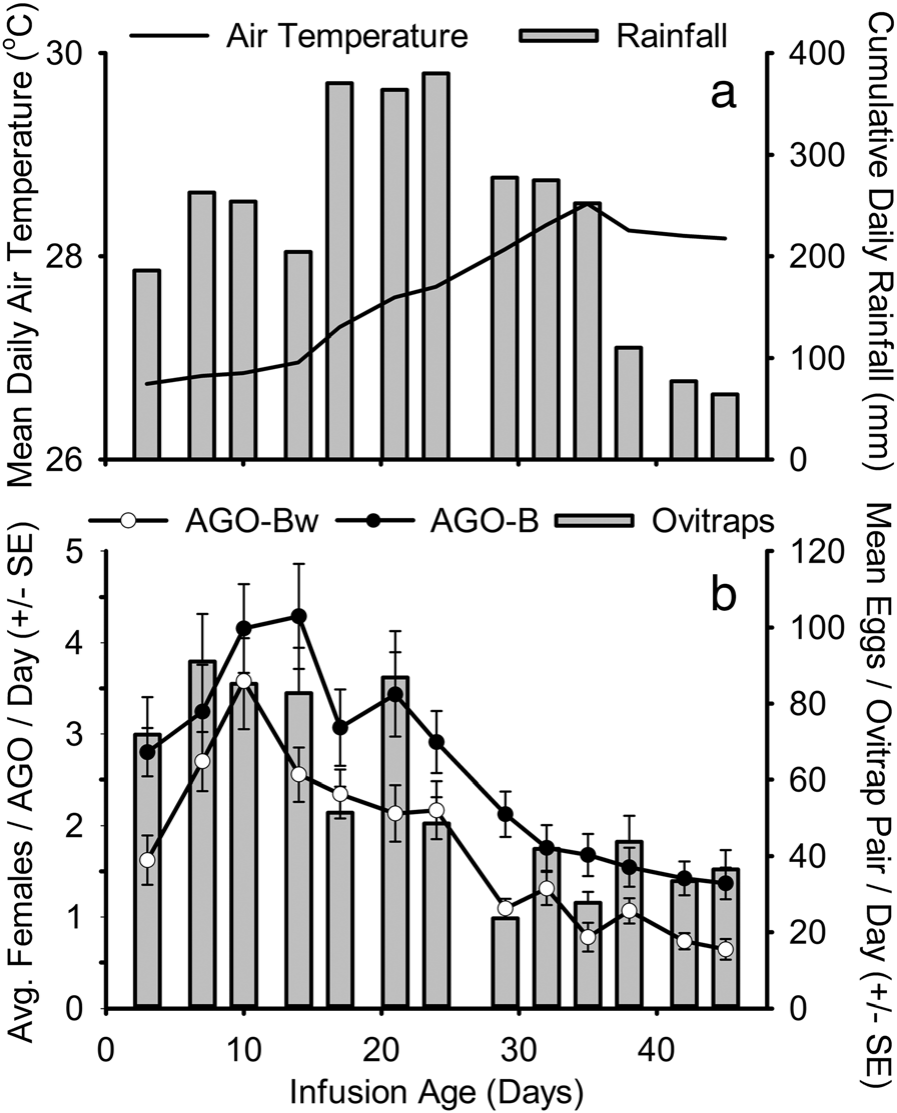
**Field comparison of the improved trap baited with infusion (AGO-B) or water (AGO-Bw).** Cumulative daily rainfall and average daily mean air temperature 8 to 28 days preceding sampling **(a)**, and the average numbers of *Aedes* eggs collected in ovitrap pairs and *Aedes aegypti* adult females collected in AGOs **(b)**, from 6 June to 21 July, 2011 (Field experiment 3).

In the first field experiment, the mean numbers of *Ae. aegypti* females collected on each collection date were on average 3.7 fold greater in the AGO-B than in the original (AGO-A) trap design (Figure 4). In the second field experiment (Figure 5), traps with a partial replacement of the infusion bait (AGO-Bp), and traps with a complete bait replacement (AGO-B) captured similar numbers of *Ae. aegypti* females on most collection dates. In the third field experiment (Figure 6), the addition of hay increased the capture rate of *Ae. aegypti* females in the AGO-B by an average of 1.6 fold, compared with traps only baited with water (AGO-Bw).

Over the course of field testing, increases in the average capture rate and the frequency of positive samples were observed in the paired ovitraps and AGO devices, coincident with increasing rainfall intensity in each sequential experiment (Table 3, Figures 4, 5 and 6). In field experiment 1, when *Ae. aegypti* abundance and rainfall were lowest, the AGO-B had a lower coefficient of variation and higher proportion of positive samples than either the AGO-A or the ovitrap pair (Table 3). Differences in sensitivity or precision between the AGO-B and other sampling devices were lower or absent in the second and third experiments. Average capture rates of adult females in AGOs were significantly correlated with cumulative rainfall preceding sampling in field experiments 1 and 2, but this relationship was marginally insignificant in the third experiment (Table 3). No significant correlations were detected between lagged rainfall and collections in the paired ovitraps (Table 3). For all three field tests, both trap design and cumulative rainfall 2 to 4 weeks prior to sampling were significant sources of variability in adult female *Ae. aegypti* collections in the AGO (Table 4). Average daily air temperature 2 to 4 weeks prior to sampling also was a significant covariate in the models for field experiments 2 and 3 (Table 4).

**Table 3:** Precision and sensitivity of ovitraps and AGOs and relationships between capture rate and rainfall

**Field experiment (n**^**a**^**)**	**Sampling device**	**Avg. specimens / trap / day (95% CI)**	**CV**^**b**^	**Avg. proportion samples positive (95% CI)**	**Spearman correlation with rain 8to28**^**c**^
1	AGO-B	1.16 (0.14)	0.91	0.89 (0.08)	0.58 *
(18)	AGO-A	0.36 (0.06)	1.25	0.55 (0.07)	0.56 *
	Ovitrap Pair	23.31 (5.77)	1.40	0.60 (0.08)	−0.27 NS
2	AGO-B	1.74 (0.58)	1.03	0.94 (0.04)	0.96 **
(7)	AGO-Bp	1.99 (0.82)	1.02	0.95 (0.05)	0.83 *
	Ovitrap Pair	37.19 (9.79)	1.02	0.89 (0.04)	0.67 NS
3	AGO-B	2.60 (0.62)	0.84	0.99 (0.03)	0.53 NS
(13)	AGO-Bw	1.75 (0.55)	0.95	0.94 (0.04)	0.53 NS
	Ovitrap Pair	55.73 (14.76)	0.91	0.98 (0.02)	0.24 NS

^a^Number of collection dates.

^b^Coefficient of variation = SD / mean.

^c^* = 0.05 > *P* ≥ 0.01, ** = 0.01 > *P* ≥ 0.001, NS = not significant (*P ≥* 0.05).

**Table 4:** Generalized linear mixed models comparing influences of AGO design and meterological conditions on capture rate

	**Exp 1. AGO-B vs AGO-A**		**Exp 2. AGO-B vs AGO-Bp**		**Exp 3. AGO-B vs AGO-Bw**	
Fixed effects^a^	F	*P*-value	F	*P*-value	F	*P*-value
trap type	310.6	<0.001	10.1	<0.01	100.6	<0.001
rain8to28	33.4	<0.001	134.8	<0.001	70.3	<0.001
temp8to28			23.3	<0.001	248.7	<0.001
Model	171.9	<0.001	50.8	<0.001	145.0	<0.001
(d.f.1, d.f.2)	(2, 1039)		(3, 402)		(3, 776)	

^a^rain8to28 = cumulative rainfall 8 to 28 days prior to sampling; temp8to28 = average daily mean air temperature 8 to 28 days prior to sampling.

### Optimal sample size calculations

**Figure 7:**
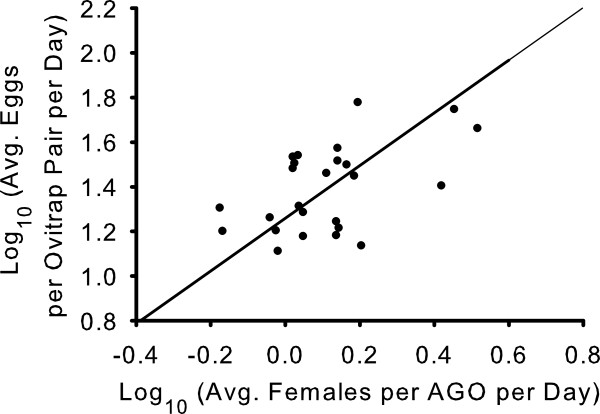
**Major axis regression comparing collections in the AGO-B and paired ovitraps.** Scatter plot of the log_10_ transformed mean numbers of adults collected per AGO per day (*m*_a_) and log_10_ transformed mean numbers of eggs collected per ovitrap pair per day (*m*_e_) for each sampling date in field experiments 1 and 2. Line represents a fitted major axis regression [Log_10_ (*m*_e_) = 1.250 + 1.306 Log_10_ (*m*_a_)].

Coefficients for the Taylor Power Law regression of data collected from paired ovitraps in the third field experiment were significantly different from coefficients derived from data collected in field experiments 1 or 2, therefore these data were excluded from the pooled data sets used to calculate minimum sample size. Taylor Power Law regression models using data pooled from the first two experiments were significant for both the AGO-B (R^2^ = 0.755, F = 70.9, df = 1, 23, *P* < 0.001) and the paired ovitraps (R^2^ = 0.722, F = 51.8, df = 1, 23, *P* < 0.001). The slope and y-intercept (95% CI) for the AGO-B were 1.832 (1.382, 2.283) and −0.118 (−0.207, -0.030), respectively. The slope and y-intercept for the paired ovitraps were 1.203 (0.855, 1.552) and 1.279 (0.783, 1.775), respectively. A major axis regression of the log_10_ transformed mean values for the first two field experiments produced a slope of 1.306 (0.520, 2.092) and y-intercept of 1.250 (1.128, 1.372; Figure 7). Minimum sample sizes calculated using coefficients from Taylor’s Power Law regressions of the pooled data sets were similar between the two sampling methods at equivalent high vector densities, but considerably fewer AGO-Bs were sufficient to estimate abundance compared with the paired ovitraps at equivalent low vector densities (Table 5).

**Table 5:** Optimal sample size calculations

**AGO-B**				**Ovitrap pair**			
***m***_***a***_	***D*** **= 0.1**	***D*** **= 0.2**	***D*** **= 0.3**	***m***_***e***_	***D*** **= 0.1**	***D*** **= 0.2**	***D*** **= 0.3**
**0.2**	383	96	43	**2.2**	3934	983	437
**0.4**	341	85	38	**5.4**	1912	478	212
**0.6**	319	80	35	**9.1**	1254	313	139
**0.8**	304	76	34	**13.3**	929	232	103
**1.0**	292	73	32	**17.8**	737	184	82
**1.2**	284	71	32	**22.6**	609	152	68
**1.4**	276	69	31	**27.6**	519	130	58
**1.6**	270	68	30	**32.9**	452	113	50
**1.8**	265	66	29	**38.3**	400	100	44
**2.0**	260	65	29	**44.0**	358	90	40
**2.2**	256	64	28	**49.8**	324	81	36
**2.4**	252	63	28	**55.8**	296	74	33
**2.6**	249	62	28	**61.9**	272	68	30
**2.8**	246	62	27	**68.2**	252	63	28
**3.0**	243	61	27	**74.7**	235	59	26

Comparison of the minimum number of sampling devices required to estimate relative abundance of *Ae. aegypti*, at three precision levels (*D*), based on expected mean females collected per AGO-B per day (*m*_a_), or expected mean eggs collected per ovitrap pair per day (*m*_e_). Expected numbers of specimens per trap per day for both devices were aligned using a fitted major axis regression [Log_10_ (*m*_e_) = 1.250 + 1.306 Log (*m*_a_)].

### Long-term efficacy of the AGO-B

At the conclusion of the field experiment 1 (62 days), the volume of infusion still remaining in AGO-B traps ranged from 5.5 to 9.0 l (mean = 74% of original volume). Cumulative rainfall during the study period was 89.9 mm. Based on the diameter of the capture chamber, it was estimated that traps could have received up to 1.8 l of water from direct exposure to rainfall over the study period. None of the traps were lost or damaged during the 62 day period. In the laboratory assay, the numbers of adult females captured, and the number of eggs collected on the PAM per captured female, were not significantly different between AGO-Bs with freshly-prepared and previously used CSs (Table 6).

**Table 6:** Capture efficiency of aged capture surfaces (CSs)

	**CS age (days)**	**Avg. per trap (95% CI)**	**F**	***P*-value**^**a**^
Adult females	0	16.4 (12.6, 20.2)	3.1	NS
	62	20.9 (17.1, 24.7)		
Eggs collected per captured female	0	15.0 (8.2, 23.8)	0.1	NS
	62	13.6 (7.2, 22.1)		

^a^NS: not significant (*P* ≥ 0.05).

## Discussion

Our objective in this study was to improve our original autocidal gravid ovitrap device so that it was a more efficacious and practical tool for vector surveillance and control. Several trap designs intermediate to the AGO-A and AGO-B were compared in order to assess sequential changes to the appearance, the trap entrance, and volume/surface area of the attractant. We also assessed the contribution of a hay infusion bait to trap performance.

Several trap devices use contrasting black and white surfaces to improve capture of *Ae. aegypti*[15,44,45]. However we found that the AGO-B was most effective when constructed entirely of black materials - increasing the visual contrast of the trap entrance by the use of a white pail lid did not improve capture efficiency. In competitive assays in the field cage, enlarging the size of the infusion reservoir and trap entrance significantly enhanced trap performance. Likely these modifications promoted orientation and entry of females approaching the trap entrance in part via a greater release of water vapor and other attractive compounds from the infusion. In laboratory tests, females were often observed making several rapid, short flights that transversed the air space directly above the entrance prior to entry. It has been suggested that a similar behaviour observed in T*oxorhynchites* spp. during oviposition (“looping flight”) allows females to detect a humidity gradient that indicates the presence of a potential oviposition site [46]. Unexpectedly, the addition of a ¾” mesh over the entrance of the AGO-B did not appear to impede entry of mosquitoes, rather it improved trap performance.

Previous assessments of infusion baits have not shown that they enhance capture of adult *Ae. aegypti* in sticky ovitraps in a natural setting [16,36]. Zang and Lei [47] observed that a Bermuda-grass infusion bait significantly increased attraction of *Ae. albopictus* adults to sticky traps in the laboratory, but not in a subsequent field test. The authors concluded that in a natural setting, the influence of infusion odor on preoviposition behavior may be overshadowed by visual stimuli. In our field cage tests, a 1:1 dilution of a week-old, anaerobically-fermented infusion made from hay (*C. nlemfuensis*) increased attraction by ≈ 2.5 fold in the AGO-A, and ≈ 2 fold in the AGO-B, relative to traps containing only tap water. Similarly addition of a hay packet to the AGO-B increased capture of female *Ae. aegypti* by an average of 1.6 fold in the third field experiment. In the field cage experiment, infusions aged in an open container (ie., *in situ*) were less attractive than infusions produced under anaerobic conditions, as previously reported [48,49].

Attraction of gravid *Ae. aegypti* females to plant-based infusions is influenced by the type and concentration of organic material used, and the length of time and conditions under which the infusion is aged [48,49]. Ponnusamy *et al.*[50] demonstrated that these factors influence the diversity and abundance of microorganisms associated with attraction. In ovitrap devices, the addition of plant material as a substrate for microbial growth (ie., *in situ* infusion) can extend the duration of olfactory attraction between replacement of the infusion bait [32]. However, as the organic material is consumed, the attractiveness of the infusion odor may diminish due to biochemical changes in the infusion resulting from succession in the microbial community. Variability in relative attractiveness over time could limit the precision of surveillance data collected using infusion-baited ovitraps. In our third field experiment, the relative difference between captures in water baited and infusion baited AGO-Bs was consistent over time, suggesting that the attractiveness of the *in situ* infusion to gravid *Ae. aegypti* females was not diminished after more than 6 weeks of use. This would indicate that changes in infusion odor are unlikely to introduce a strong bias in surveillance data collected with the AGO-B during long-term use.

Several studies have reported that waters containing (or previously containing) conspecific larvae can enhance oviposition by *Ae. aegypti* females, in comparison with distilled or tap water [51-53]. Semiochemicals released by conspecific larvae or bacteria associated with the larvae may also act as attractants to gravid females [54,55]. In the current study, adding up to 500 larvae to the infusion attractant did not significantly enhance attraction to the AGO-B. Benzon and Apperson [54] observed peak oviposition by *Ae. aegypti* in water that had previously held 1 conspecific larva per ml; a much greater density than the one assayed in this study (0.0625 larvae per ml). However, the purpose of our experiment was to determine if colonization of the AGO-B by the progeny of captured females could influence the attractiveness of the trap. In practice, the density of larvae in an AGO-B is expected to be far less than 1 larva per ml. Though the density was not quantified, none of the traps that were found positive for *Ae. aegypti* immatures after extended field use (62 days) appeared to hold more than 100 larvae or pupae (<0.02 larvae / ml).

There are conflicting descriptions of the response of *Ae. aegypti* adult populations to rainfall. While some studies have observed that adult abundance and oviposition activity are positively associated with precipitation [56,57], others have failed to detect a relationship [13,58] or have found other meteorological factors to be more predictive [59]. Some of this discrepancy may be attributed to local or regional differences in the relative importance of aquatic habitats receiving water directly from human activities versus container habitats filled by rainfall [60]. In metro San Juan, PR, both human-managed and rain-filled containers are important for *Ae. aegypti* development [40]. In the current study, there were significant associations between changes in the capture rate in the AGO-B and cumulative rainfall 8 to 28 days preceding sampling, consistent with a previous study at the same location where numbers of *Ae. aegypti* collected in BG-sentinel traps were positively correlated with an 8 to 21 day lag in cumulative rainfall [40]. Interestingly, no relationship was detected between rainfall and egg collections in the paired ovitraps. The strength of the relationship between rainfall and adult captures in the AGO-B was not consistent among the three field tests; the strongest association was evident in the first two field tests when rainfall intensity and frequency were lower. Similar seasonal variability in the strength of the response to rainfall has been reported in other populations of *Ae. aegypti*[60,61].

At lower vector densities (field experiment 1), the AGO-B provided a more sensitive measure of adult activity, relative to paired ovitraps, with fewer AGO-Bs required to monitor *Ae. aegypti* populations with an equivalent level of precision. These results suggest that the AGO-B might be a more practical surveillance tool for programs designed to detect the introduction and spread of *Ae. aegypti* to new areas (eg. [62]). However at higher vector densities (field experiments 2 and 3), the AGO-B and paired ovitraps had equivalent precision and sensitivity. An inverse relationship was reported by Faccinelli *et al.*[63], who observed a similar or higher level of precision and sensitivity using a sticky ovitrap, compared with conventional ovitraps, for sampling *Ae albopictus* at moderate to high densities, but not at low densities. Other field assessments with sticky ovitrap devices have reported them to be equally or less sensitive for detecting the presence of *Ae. aegypti*, relative to conventional ovitrap sampling [34,57].

Although the absence of a common, independent measure of the underlying density of adult vectors makes it difficult to make direct comparisons with previous field studies evaluating similar passive trapping devices, our field data suggest that the AGO-B has a relatively high proficiency to attract and capture *Ae. aegypti* females. Most other field studies with sticky ovitraps have observed average capture rates of less than 0.2 *Ae. aegypti* females per trap per day [13,16,18,19,34,35,64-66], although collection rates of greater than 3 females per trap per day have been reported [67]. These sticky ovitraps are much smaller than the AGO-B, with a maximum capacity of a few hundred ml to 2 l of water or infusion bait. This apparent advantage in the capture rate of the AGO-B over similar devices could be due to the larger infusion reservoir, as evidenced by the results of the field cage studies, where the AGO-B captured several fold more adult females than a modified AGO-A with a similar appearance and trap entrance diameter, but smaller infusion reservoir.

## Conclusions

We achieved a substantial inprovement in capture rate by modifying our original autocidal gravid ovitrap. Results of the three field tests validate that capture efficiency and relative attractiveness of the modified trap (AGO-B) can be maintained by periodic (≈ every 6–8 weeks) replacement of the capture surface, hay substrate and water lost to evaporation. Our original device (AGO-A) had previously been found to significantly reduce the abundance of potentially infected (gravid and parous) females of *Ae aegypti* when used concurrently with source reduction and larvicide applications in an area wide vector control program (CDC, unpublished data). We expect that the improved device (AGO-B) will have an even greater impact on vector populations than observed with the AGO-A. The low cost (materials ≈ $6.50 per trap) and its ability to be used for extended periods without maintenance could permit long-term, large scale deployment of these devices. Addionally, the high sensitivity for detecting the presence of *Ae. aegypti* at low density also demonstrates the potential value of the AGO-B for monitoring re-infestation or recovery following an area wide application of integrated vector control efforts. Future research efforts will include a robust, community scale assessment of the AGO-B as a vector control and surveillance tool*.*

## Abbreviations

AF: Anaerobically-fermented; AGO: Autocidal gravid ovitrap; CS: Capture surface; ED: Entrance diameter; GLMM: Generalized linear mixed model; GO: Gravid ovitrap; PAM: Polyacrylamide copolymer hydrogel.

## Competing interests

The authors declare that they have no competing interests. This research was fully funded by the US Centers for Disease Control and Prevention (CDC) which holds a provisional patent for the devices described in this manuscript (autocidal gravid ovitraps).

## Authors’ contributions

AJM designed and implementated laboratory assays and field investigations, conducted data analysis and interpretation, and manuscript writing. MA conducted laboratory and field experiments, contributed with trap design improvements. RB conceived the study, designed experiments, conducted data analysis and interpretation, and manuscript writing. All authors read and approved the final version of the manuscript.
